# Anti-vascular endothelial growth factor therapy-induced glioma invasion is associated with accumulation of Tie2-expressing monocytes

**DOI:** 10.18632/oncotarget.1893

**Published:** 2014-04-11

**Authors:** Konrad Gabrusiewicz, Dan Liu, Nahir Cortes-Santiago, Mohammad B. Hossain, Charles A. Conrad, Kenneth D. Aldape, Gregory N. Fuller, Frank C. Marini, Marta M. Alonso, Miguel Angel Idoate, Mark R. Gilbert, Juan Fueyo, Candelaria Gomez-Manzano

**Affiliations:** ^1^ Department of Neuro-Oncology, The University of Texas MD Anderson Cancer Center, Houston, Texas, USA; ^2^ Department of Pathology, The University of Texas MD Anderson Cancer Center, Houston, Texas, USA; ^3^ Institute of Regenerative Medicine, Wake Forest University, Winston-Salem, North Carolina, USA; ^4^ Department of Medical Oncology, University Hospital of Navarra, Pamplona, Spain; ^5^ Department of Pathology, University Hospital of Navarra, Pamplona, Spain; ^6^ Department of Genetics, The University of Texas MD Anderson Cancer Center, Houston, Texas, USA

**Keywords:** Brain tumors, antiangiogenesis, Tie2, tumor microenvironment, monocyte

## Abstract

The addition of anti-angiogenic therapy to the few treatments available to patients with malignant gliomas was based on the fact that these tumors are highly vascularized and on encouraging results from preclinical and clinical studies. However, tumors that initially respond to this therapy invariably recur with the acquisition of a highly aggressive and invasive phenotype. Although several myeloid populations have been associated to this pattern of recurrence, a specific targetable population has not been yet identified. Here, we present evidence for the accumulation of Tie2-expressing monocytes/macrophages (TEMs) at the tumor/normal brain interface of mice treated with anti-VEGF therapies in regions with heightened tumoral invasion. Furthermore, we describe the presence of TEMs in malignant glioma surgical specimens that recurred after bevacizumab treatment. Our studies showed that TEMs enhanced the invasive properties of glioma cells and secreted high levels of gelatinase enzymatic proteins. Accordingly, Tie2^+^MMP9^+^ monocytic cells were consistently detected in the invasive tumor edge upon anti-VEGF therapies. Our results suggest the presence of a specific myeloid/monocytic subpopulation that plays a pivotal role in the mechanism of escape of malignant gliomas from anti-VEGF therapies and therefore constitutes a new cellular target for combination therapies in patients selected for anti-angiogenesis treatment.

## INTRODUCTION

Despite therapeutic advances over the last decade, the diagnosis of glioblastoma, the most frequent and aggressive type of primary brain tumor, is associated with a median overall survival of 15-18 months and with a 5-year survival rate of less than 5% [1, 2]. Even if treatment with the current standard of care for glioma patients, which consists of surgery, the DNA-alkylating drug temozolomide (TMZ), and radiotherapy, is initially successful, nearly all malignant gliomas eventually recur [3]. On the basis of reports of response rates [4, 5], the U.S. Food and Drug Administration approved in 2009 the use of bevacizumab, a human recombinant monoclonal antibody against vascular endothelial growth factor (VEGF), for the treatment of recurrent glioblastoma. There is a strong rationale for using anti-angiogenic therapy for these tumors, as they are highly vascularized—microvascular proliferation or necrosis (or both) is an essential diagnostic criterion for glioblastoma [6]—and several reports have confirmed that blocking tumor angiogenesis inhibits tumor growth [7-9]. Beside bevacizumab, additional anti-angiogenic monotherapies have been or are being tested in trials for patients with recurrent glioblastoma, such as the VEGF trap aflibercept, which is a recombinant fusion protein that inhibits both VEGF and placental growth factor, and the VEGF receptor (VEGFR) inhibitor cediranib. However, current evidence from experimental and clinical studies suggests that glioblastomas can recur after these therapies [10, 11] as highly aggressive tumors characterized by enhanced invasiveness and resistance to all currently available therapies [9, 12-16]. The mechanisms underlying the development of this invasive phenotype are not fully understood.

Among the studies addressing the fact that recurring tumors can be even more invasive and resistant to treatment, several have addressed the contribution of tumor/stroma interactions to the development of inherent refractoriness or acquired resistance to anti-angiogenic therapy. In murine preclinical models of lung cancer and lymphoma, refractoriness to anti-VEGF therapy was linked to tumor infiltration of CD11b^+^Gr1^+^ myeloid cells [17]. In clinical trials with glioblastoma patients treated with aflibercept, a decrease in circulating VEGFR1^+^CD14^+^ monocytes was associated with improved response [18]. In addition, autopsy results of patients who had been treated with anti-angiogenic agents, including bevacizumab and cediranib, revealed that higher number of CD11b^+^ cells in bulk and infiltrative tumors correlated with poor overall survival [19, 20]. However, the cell surface markers that were used to identify those cell subsets can also be used to identify a broad range of myeloid cells, and a specific monocytic population associated with the heightened invasion observed with anti-angiogenic therapy has not been completely characterized.

Tie2-expressing monocytes (TEMs) are a subpopulation of circulating blood monocytes that have recently been identified in several syngeneic and xenograft tumors (such as gliomas, sarcomas, and kidney, colon, pancreatic, and lung cancers) and in peripheral blood of humans and mice [21-23]. TEMs are preferentially recruited to tumors, where they constitute a prominent monocyte population distinct from tumor-associated monocytes [22-24]. We explored whether TEMs contribute to the refractoriness of gliomas to anti-angiogenic treatment. Our findings suggested that the presence of TEMs is associated with the development of an invasive glioma phenotype that is resistant to anti-VEGF therapy.

## RESULTS

### TEMs are overrepresented at the invasive front of tumors that had been treated with anti-VEGF agents

**Figure 1:**
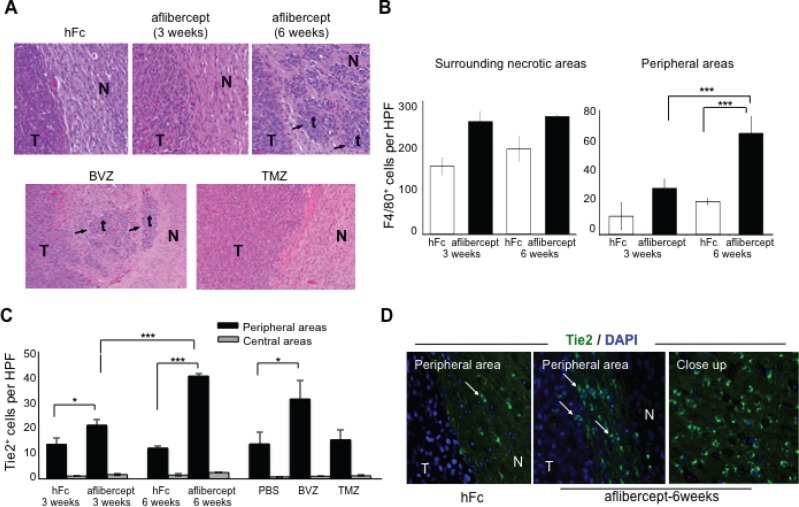
Accumulation of tumor-infiltrating microglia/macrophages and Tie2^+^ cells at the tumor edge of intracranial U-87 MG glioma-bearing mice after anti-VEGF treatment (A) Anti-angiogenic treatment induced invasive tumor outgrowth in the malignant glioma model. Tumor sections from mice treated, as indicated in Methods section, with hFc (control), aflibercept (for 3 weeks or 6 weeks), bevacizumab (BVZ), or TMZ were stained with Harris hematoxylin and eosin. Arrows point to tumor nodules and satellitosis in sections from tumor-bearing mice that received aflibercept for 6 weeks or bevacizumab. No invasive glioma features (N, normal brain; T, tumor; t, tumor nodule) were observed in animals treated with hFc, aflibercept for 3 weeks, or TMZ. Magnification: upper row (20x), lower row (10x). (B) Quantification of F4/80^+^ cells in surrounding necrotic tumor areas and in peripheral tumor/normal brain edges in mice treated with hFc or aflibercept as indicated. F4/80^+^ cells were counted with a high-power field (HPF) (200x). Data are presented as mean ± SD (*n* = 4-5 animals per group). ^***^ *P* < 0.001. (C) Quantification of Tie2^+^ cells at glioma tumor edge and at the tumor center from mice treated with hFc for 3 weeks or 6 weeks, aflibercept for 3 weeks or 6 weeks, phosphate-buffered saline (PBS; a control), bevacizumab, or TMZ. Tie2^+^ cells were counted with a high-power field (HPF) (200x). Data are presented as mean ± SD (*n* = 4-5 animals per group). ^*^ *P* < 0.05; ^***^ *P* < 0.001. (D) Representative images of Tie2 staining of sections from mice treated with hFc or aflibercept (6 weeks). Pictures show merged fluorescent Tie2 (green) and DAPI (blue). Arrows indicate Tie2^+^ cells. N, normal tissue; T, tumor. Magnification: 200x; closeup, 400x.

We previously described that U-87 MG intracranial human xenograft-bearing mice treated with aflibercept long term (6 weeks) had a longer median survival time than did mice treated for only 3 weeks [25]. We also reported that greater glioma invasion was associated with the 6-week schedule, as characterized by the presence of “satellitosis” or “secondary structures” involving tumor cell aggregations in perivascular regions and in Virchow-Robin spaces. These structures were easily identified because this animal glioma model does not display invasive features [25, 26]; accordingly, the gliomas of human Fc region (hFc)-treated animals were well delineated and exhibited no signs of invasion. In the current study, we observed that this invasive glioma phenotype was also present in animals that had been treated with the anti-VEGF antibody bevacizumab, as previously reported [12]. However, the invasive glioma phenotype was not observed in animals treated with TMZ (Figure 1A). To understand the role of stroma on invasive glioma growth, we analyzed first for the presence of microglia/macrophages, which have been reported to be associated with adaptive resistance to anti-angiogenic therapy [27, 28]. Using the F4/80 or Iba1 markers for microglia/macrophages, we observed, as expected, abundant F4/80^+^ and Iba1^+^ cells in surrounding necrotic areas. In addition, we observed an increase in the presence of this population in aflibercept-treated animals compared with hFc-treated animals, although no notable differences were observed in animals treated for 3 weeks (for non-invasive gliomas) or 6 weeks (for invasive gliomas) with this drug (Figure 1B). Our observations suggested that this perinecrotic myeloid population was not associated with the invasive phenotype induced by anti-VEGF therapy.

Of interest, administration of aflibercept for 6 weeks was associated with significantly more F4/80^+^ cells in the tumor/normal brain interface than was treatment with aflibercept for 3 weeks (Figure 1B, *P* < 0.001). This dramatic accumulation of microglia/macrophages in the tumor/normal brain interface was corroborated using another marker of myeloid lineage activity, Iba1. An overrepresentation of Iba1^+^ cells was evident in the tumor/normal brain interface in the two anti-angiogenic treatment regimens that resulted in heightened glioma invasion (6-week treatment with aflibercept or bevacizumab) compared with the treatment regimens not linked to an invasive glioma phenotype (hFc, 3-week aflibercept, and TMZ) (Supplemental Figure S1). Together, these results suggested that an increased presence of microglia/macrophages in the tumor/normal brain interface is associated with the invasive pattern observed in glioma-bearing mice treated with anti-angiogenic agents.

A specific population of monocyte/macrophages, TEMs, are a distinctive subpopulation of tumor-infiltrating CD11b^+^ myeloid cells [23, 29]. To investigate whether TEMs contribute to the heightened invasiveness of glioma after anti-VEGF therapy, we initially immunostained for Tie2 in human glioblastoma xenografts. Tie2^+^ cells were present along the periphery of the tumor (Figure 1C,D) and barely detectable in surrounding necrotic areas, in contrast with the Iba1^+^ or F40/80^+^ cells that were present in both locations (Figure 1B, Supplemental Figure S1). Of interest, the number of Tie2^+^ cells was significantly greater in the brains of the animals with an invasive glioma phenotype (treated with aflibercept during 6 weeks or bevacizumab) than in those harboring non-invasive tumors (treated with vehicle, TMZ, or the short schedule of aflibercept) (Figure 1C, *P* < 0.001, aflibercept-6 weeks versus aflibercept-3 weeks).

**Figure 2:**
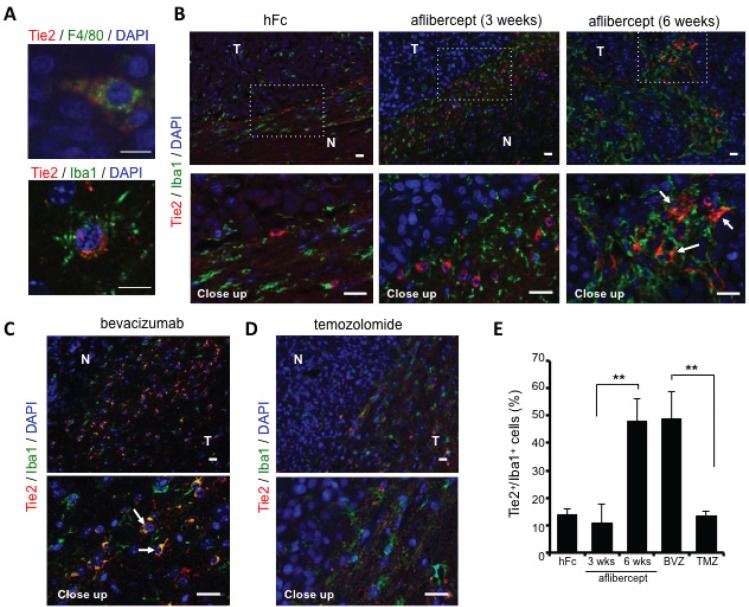
Over-representation of TEMs at the invasive front of tumors treated with anti-VEGF agents (A) Double immunofluorescence revealed co-localization of Tie2 (red) and F4/80 (green, top) or Iba1 (green, bottom) using confocal microscopy. DAPI was used for nuclear staining (blue). Scale bar = 10 μm. (B) Representative fluorescence images of Tie2 (red) and Iba1 (green) staining in brains of U-87 MG-bearing mice that received hFc or aflibercept (3 weeks or 6 weeks). DAPI was used for nuclear staining (blue). White arrows point to Tie2^+^Iba1^+^ cells. N, normal brain; T, tumor. Scale bar = 20 μm for both top and bottom rows. (C-D) Representative pictures of Tie2/Iba1 immunofluorescence on brain tumor slides from animals treated with bevacizumab (C) or TMZ (D). White arrows indicate Tie2^+^Iba1^+^ cells. DAPI was used for nuclear staining (blue). Scale bar = 20 μm for both top and bottom rows. (E) Quantification of percentage of Tie2^+^/Iba1^+^ cells at tumor periphery of mice treated with hFc, aflibercept (3 weeks or 6 weeks), bevacizumab (BVZ), or TMZ. Data are presented as percentage ± SD of Tie2^+^/Iba1^+^ cells among Iba1^+^cells (*n* = 3-4 animals per group). ^**^ *P* < 0.01.

Confocal microscopy analysis revealed co-localization of Tie2 expression with the Iba1 and F4/80 monocyte/macrophage markers, thereby identifying monocytic lineage cells with heterogeneous morphology (e.g., spindle, rod, and amoeboid shapes) and small nuclei characteristic of “activated” microglia/macrophages (Figure 2A). We detected more Tie2^+^Iba1^+^ cells at the tumor edge in brains of mice treated with aflibercept during 6 weeks or bevacizumab (invasive pattern) than in mice treated with hFc, 3-week aflibercept, or TMZ (non-invasive pattern) (Figure 2B-E). Our observations supported the notion that infiltrative TEMs are associated with the heightened invasion that occurs with anti-angiogenic therapy.

To determine whether the Tie2^+^ myeloid cells present in the tumor/normal brain edge upon anti-VEGF therapy are an M2 polarized population [22], we performed double immunofluorescence staining with Tie2 and Arg1 [30]. Our observation of an accrual of Tie2^+^Arg1^+^ cells at the invasive front of tumor-bearing mice treated anti-VEGF agents (Supplemental Figure S2) further supported an association between TEMs and invasiveness upon anti-VEGF therapy.

### TEMs enhance the invasiveness of glioma cells

**Figure 3:**
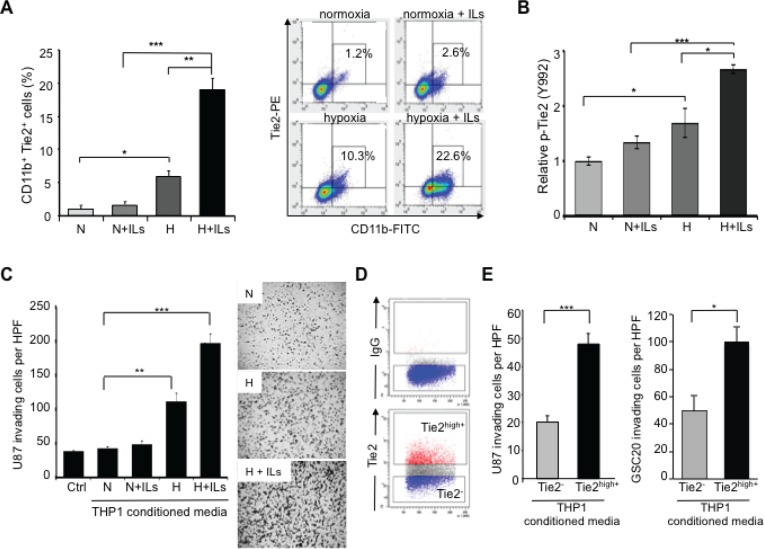
TEMs induce an invasive glioma cell phenotype (A) Quantification of CD11b^+^Tie2^+^ subpopulations in THP-1 monocytic cells exposed to normoxia alone (N), hypoxia (H) alone, or co-stimulated with IL4 and IL13 (ILs). Left panel: Data are presented as mean ± SD from three independent experiments. ^*^P < 0.05, ^**^P < 0.01, ^***^P < 0.001. Right panel: Representative dot plot illustrating the percentage of Tie2^+^CD11b^+^ cells after the indicated culture treatments. (B) Determination of Tie2 phosphorylation levels in THP-1 monocytic cells exposed to normoxia alone, hypoxia alone, or co-stimulated with ILs. Data represent relative fluorescence determined by normalizing phospho-Tie2 fluorescence unit levels to the total Tie2 fluorescence unit levels. ^*^P < 0.05, ^***^P < 0.001. (C) Invasion assay highlighted the role of TEMs in the invasive phenotype of gliomas. Conditioned medium was collected from THP-1 cells subjected to normoxia or hypoxia in the presence or absence of ILs and placed in the bottom well of a modified Boyden chamber. U-87 MG cells were plated in the top Matrigel-coated transwells. Left panel: Data are presented as mean ± SD of invading U-87 MG cells per HPF (200x). ^**^P < 0.01, ^***^P < 0.001. Right panel: Representative images of crystal violet–stained U-87 MG cells that invaded the Matrigel layer (magnification, 50x). (D) Representative dot plot analysis for the identification of Tie2^+^ and Tie2^-^ cell populations from THP-1 monocytic cells that were exposed to hypoxia and ILs. Selected populations were sorted and conditioned medium was collected. (E) U-87 MG and GSC20 invasion properties were measured using a modified Boyden chamber assay as explained in (C), where conditioned media were obtained from sorted Tie2^+^ and Tie2^-^ THP-1 cultures. Data are presented as mean ± SD number of invading glioma cells per microscopic field (200x) from three independent experiments. ^***^ *P* < 0.001, ^*^ *P* < 0.05.

Because we observed an association between the presence of TEMs and heightened invasion upon anti-VEGF therapy, we next examined the role of TEMs in tumor invasion. To overcome the difficulties associated with monocytic cultures, we generated an in vitro model based on the THP-1 monocytic cell line by enriching this culture for the presence of TEMs. Thus, we treated THP-1 cells with interleukins 4 and 13 (IL4 and IL13) under hypoxic conditions to induce M2 polarization and Tie2 expression [31, 32]. Under these experimental conditions, we observed a significant increase in the number of Tie2^+^CD11b^+^ cells within the THP-1 population compared with parental or normoxia-cultured cells (Figure 3A, *P* < 0.001). In addition, we detected an increase in the relative presence of phosphorylated Tie2 protein, via a cell-based enzyme-linked immunosorbent assay (ELISA), under hypoxic conditions compared with normoxic conditions (Figure 3B, *P* < 0.05) that was further enhanced in THP-1 cells exposed to IL4 and IL13 (Figure 3B, *P* < 0.001, 2-tailed Student *t*-test).

In addition we exposed glioma cells to conditioned medium from THP-1 cells with different levels of Tie2^+^ monocyte enrichment, as explained above. Compared with the group treated under normoxia and without ILs, the number of invading U-87 MG cells was higher in the group cultured under hypoxic conditions (*P* < 0.01) and was even higher when ILs were added (Figure 3C, *P* < 0.001). Of interest, the expression level of Tie2 in the monocytic cultures was significantly correlated with the invasive properties of U-87 MG cells incubated with conditioned medium from these cultures (Pearson's *r* = 0.98; *P* < 0.05).

To further confirm the role of TEMs in promoting an invasive phenotype of glioma cells, we sorted Tie2^high+^ and Tie2^-^ subpopulations from THP-1 monocytic cells (Figure 3D). Similar to the previous experiment, glioma invasiveness through Matrigel was significantly higher with conditioned medium from Tie2^high+^ monocytic cells than with conditioned medium from Tie2^-^ monocytic cells (Figure 3E, *P* < 0.001, 2-tailed Student *t*-test). These results were confirmed using GSC20 glioma stem cell cultures (Figure 3E, *P* < 0.05, 2-tailed Student *t*-test). These data highlight the superior potential of TEMs versus other monocytic populations to induce invasion of glioma cells.

### TEMs are a major source of MMP9 secretion and activity

**Figure 4:**
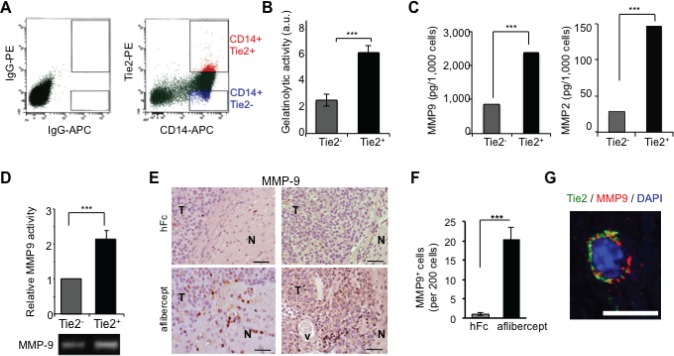
TEMs are a major source of gelatinase activity and MMP9 secretion (A) Enriched monocyte population isolated from PBMCs of healthy donors was doubly stained for CD14 and Tie2 and sorted as Tie2^+^ (CD14^+^Tie2^+^) cells or Tie2^-^ (CD14^+^Tie2^-^) cells using FACS. Shown is a representative dot plot analysis. (B) Quantification of gelatinolytic activity present in conditioned medium of sorted CD14^+^ Tie2^+^ and CD14^+^ Tie2^-^ cells obtained from the enriched monocytic population present in PBMCs. Data are represented as mean ± SD from three independent experiments. ^***^ *P* < 0.001. (C) Quantification of MMP9 and MMP2 levels present in conditioned medium of sorted CD14^+^Tie2^+^ and CD14^+^Tie2^-^ cells obtained from the enriched monocytic population present in PBMCs. Data are presented as mean ± SD from three independent experiments. ^***^ *P* < 0.001. (D) Conditioned medium from Tie2^+^ and Tie2^-^ monocytic subsets analyzed by zymography revealed higher MMP9 activity in conditioned medium from Tie2^+^ monocytic cultures than in Tie2^-^ monocytic cultures sorted from THP1 cultures. Data represent mean ± SD from three independent experiments. ^***^ *P* < 0.001. (E) MMP9 detection in sections of brains from U-87 MG xenograft-bearing mice treated with aflibercept (6-week schedule). Note the presence of MMP9 immunoreactivity in infiltrative areas of tumors from mice treated with the anti-VEGF agent. N, normal tissue; T, tumor; v, vessel. Scale bar = 50 μm. (F) Quantification of MMP9 positive cells in sections of brains from U-87 MG xenograft-bearing mice treated with aflibercept (6-week schedule). Data are represented as mean ± SD of MMP9^+^ cells per 200 cells. (G) Confocal z-stack image of Tie2 (green) and MMP9 (red) double immunofluorescence in sections from U-87 MG xenografts treated with aflibercept (6 weeks). DAPI was used for nuclear staining (blue). Scale bar = 10 μm.

Because our results demonstrated that conditioned medium from TEMs are responsible for enhancing the invasive properties of glioma cells, we analyzed whether this subpopulation is a source of tumor-remodeling activity. To address this aim, we obtained monocyte-enriched human peripheral blood mononuclear cells (PBMCs) that were sorted for CD14^+^Tie2^+^ and CD14^+^Tie2^-^ cell subpopulations (Figure 4A). We then collected the conditioned medium from both populations and performed a gelatinase activity assay, which demonstrated significantly greater median enzymatic activity in Tie2^+^ than Tie2^-^ monocyte conditioned medium (Figure 4B; *P* < 0.001). The levels of expression of the most significant gelatinase enzymes in gliomas, matrix metalloproteinases 9 and 2 (MMP9 and MMP2) [33], were also greater in the conditioned medium of CD14^+^Tie2^+^ cells than that of CD14^+^Tie2^-^ cells (Figure 4C; *P* < 0.001 for each), although the MMP2 levels were in the low range of detection. These data suggested that TEMs are a major cellular source of MMP9. This result was corroborated using the in vitro experimental THP-1 model. We observed an increased in MMP9 activity via zymography assay in experimental M2-polarized THP-1 cells (Figure 4D, *P* < 0.001).

To validate in vivo the described results, we performed MMP9 immunostaining of brain tissue sections from human glioma U-87 MG-intracranial bearing mice treated with aflibercept for 6 weeks and then presenting an invasive phenotype. In treated mice, staining revealed MMP9^+^ cells at the tumor edge and peripheral invasive tumor nodules with rod or amoeboid shapes characteristic of “activated” microglia/macrophages, which were barely observed in control animals (Figure 4E,F; Supplemental Figure S3). Consistent with our in vitro data, staining revealed that MMP9 co-localized with Tie2^+^ cells in aflibercept-treated animals, as assessed by double immunofluorescence and analysis of confocal z-stack images (Figure 4G; Supplemental Figure S3).

### Accumulation of TEMs in human malignant glioma surgical specimens upon anti-VEGF therapy

**Figure 5:**
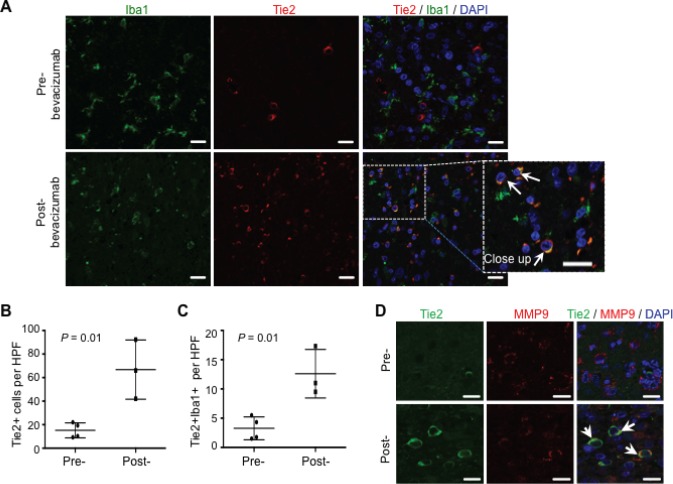
TEMs can be detected in human surgical glioblastoma specimens after bevacizumab therapy (A) Glioblastoma specimens after treatment with standard chemotherapy (pre-) or with bevacizumab (post-) were analyzed for Iba1 (green) and Tie2 (red) expression. DAPI was used for nuclear staining (blue). Scale bars = 20 μm. Note the over-representation of TEMs (Tie2^+^Iba1^+^ cells) in the post-bevacizumab tumor. White arrows indicate the presence of Tie2^+^Iba1^+^ cells. Scale bars = 20 μm. (B) Quantification of Tie2^+^ cells in surgical human glioblastoma recurring after standard therapy (Pre-; *n* = 4) or after bevacizumab (Post-; *n* = 3). Data are represented as mean ± SD of Tie2^+^ cells present in a HPF. (C) Quantification of Tie2^+^Iba1^+^ double positive cells in surgical human glioblastoma recurring after standard therapy (Pre-; *n* = 4) or after bevacizumab (Post-; *n* = 3). Data are represented as mean ± SD of Tie2^+^ cells present in a HPF. (D) Tie2^+^ cells overexpressed MMP9, as indicated with the double immunofluorescence for Tie2 (green) and MMP9 (red) expression. DAPI was used for nuclear staining (blue). White arrows indicate the presence of Tie2^+^MMP9^+^ cells. Scale bar = 20 μm.

Analysis of surgical glioblastoma specimens from patients treated with bevacizumab showed an identical distribution of this Tie2^+^Iba1^+^ cellular population in the tumor periphery. Thus, TEMs were observed at the advancing edge of the tumor after bevacizumab treatment, as assessed by double Tie2/Iba1 immunostaining and confocal microscopy (Figure 5A). Comparative studies on the presence of Tie2^+^ cells in surgical samples obtained after recurrence to standard therapy (temoradiation; *n* = 4) showed a significant increase in the number of Tie2^+^ cells when compared to those detected in surgical samples obtained upon bevacizumab therapy (*n* = 3)(Figure 5B, *P* = 0.01, Student t-test). Of further clinical relevance, we detected a significant overrepresentation of TEMs in surgical specimens upon bevacizumab therapy (Figure 5C, *P* = 0.01, Student t-test). Furthermore, in agreement with our data obtained in the animal model, confocal microscopy analysis confirmed that Tie2^+^ cells expressed MMP9 (Figure 5D). To our knowledge, this is the first evidence for the presence of TEMs in surgically excised malignant gliomas, and for the association of TEMs with the glioma recurrence after anti-VEGF therapy.

## DISCUSSION

Our findings suggested that the presence of TEMs is associated with the development of an invasive glioma phenotype that is resistant to anti-VEGF therapy. This specific Tie2^+^ monocytic population was present in tumor infiltrative areas and displayed significantly greater tumor-remodeling activity than Tie2^-^ monocytic cells did, establishing a pro-invasive environment for glioma cells.

Malignant glioma is characterized by a robust angiogenic component and, although it is not a metastatic disease, it exhibits a locally invasive pattern. The regulation of invasiveness and angiogenesis seems to be tightly interdependent: co-option of normal blood vessels by infiltrating glioma cells has been shown to be instrumental to the invasiveness of malignant glioma and to be a precursor to neovascularization [9, 34-36]. Preclinical and clinical data, including those from our studies and other groups' studies, have shown that anti-angiogenic therapy might result in an adaptive phenotype characterized by enhanced invasiveness [12, 16, 25, 37, 38]. Several explanations for this switch towards invasion have been proposed, including the contribution of the tumor microenvironment in recruiting hematopoietic cells into the tumor site [27].

We observed an over-representation of monocytic populations in the bulk of the tumors of mice treated with aflibercept, mainly in surrounding necrotic areas, compared to control (untreated) animals. However, this phenomenon was unrelated to the presence of an invasive pattern, as it was observed after the two different treatment schedules of aflibercept: a short (3-week) schedule that did not result in increased invasiveness, and a long (6-week) schedule that resulted in satellitosis and perivascular infiltration. An increase in the monocyte/macrophage population associated with anti-angiogenic therapy has been reported [18] [20], although not specifically in relation to increased invasion. Autopsy results of brain specimens revealed that the presence of CD68*^+^*, CD163*^+^*, and CD11b*^+^* cells correlated with poor overall survival among patients who first received anti-angiogenic therapy at recurrence [20]. In a clinical trial of patients with malignant gliomas who were treated with aflibercept, a decrease in VEGFR1*^+^*CD14*^+^* monocytes from baseline to 24 hours was associated with better response [18].

Of interest, we observed a significant increase in the number of F4/80^+^ or Iba1^+^ cells at the tumor/normal brain edge in tumors exhibiting invasion upon anti-VEGF therapy compared to tumors from control mice and TMZ-treated mice. Autopsy data derived from patients who had been treated with cediranib showed that the tumors exhibited a more infiltrative phenotype with an increase of infiltrative CD68^+^ myeloid cells than in patients left untreated or treated with chemotherapy [19]; however, the researchers did not follow up on this association and did not characterize this myeloid population. We report here the striking association of the enhanced invasive tumor pattern upon anti-VEGF therapy and the presence of TEMs. This phenotype was observed upon treatment with two anti-VEGF antibodies, bevacizumab and aflibercept, but it was not present after treatment with TMZ, the standard chemotherapy for malignant gliomas. Although some researchers associate anti-VEGF therapy with increased local invasion and distant metastasis, the most supportive experiment of the specificity of this relationship was reported by Paez-Ribes and colleagues [16], who demonstrated that genetic disruption of the VEGF/VEGFR2 pathway produced a similar adaptive response as blocking the VEGF/VEGFR2 association using small molecules or antibodies. Thus, the results we obtained in our study could be common to therapies that not only trap VEGF but also decrease VEGFR activity or VEGFR/VEGF binding.

TEMs might be detected in blood of healthy donors, but they have not been observed in normal, non-neoplastic tissues [21-23]. TEMs have been found in syngeneic or xenograft tumor mouse models [21-23]. Their presence in surgically resected human specimens has not been yet fully explored; our report is the first to show that this population might be detectable in surgically resected malignant gliomas. Although the number of TEMs within a tumor appears to be relatively small [29], loss-of-function studies using “suicide gene” approaches have shown that depletion of TEMs specifically can block tumor growth [9, 21]. However, no previous report has associated the heightened invasion observed after anti-angiogenic therapy with the over-representation of TEMs.

TEMs exhibit a more prominent glioma invasive phenotype than non-TEMs do, as we observed using glioma/monocyte co-culture conditions. We also discovered that TEMs exhibited greater gelatinase activity and production of MMP9 and MMP2. This finding was consistent with data from a clinical trial with aflibercept, in which tumor progression was associated with increased levels of circulating MMP9 [18]. However, the roles of MMP9 and MMP2 in the heightened invasion upon anti-angiogenic therapy might be only one force behind this phenomenon, and TEMs could secrete other tumor-remodeling molecules that contribute to the adaptive response [22, 39]. For that reason, the identification of TEMs as a distinguishable population responsible for the heightened invasion upon anti-angiogenesis strategies suggests a more successful target for glioma therapy.

Another possible therapeutic approach might be derived from studies on the signals responsible for tumor recruitment of TEMs after VEGF therapy, and probably also accountable for the specific distribution of this myeloid subpopulation within the tumor edge. Recruitment of this subpopulation seems to be governed by signals other than tumor-associated monocytes. CC chemokine ligand 2 (CCL2) is a major chemoattractant for tumor-associated monocytes; however, TEMs do not express the receptor CCR2 on their surface [40]. Several cytokines, including angiopoietin 2, and chemokines CCL3, CCL5, and CCL8, have been reported as chemoattractant molecules for TEMs [40]. Detailed descriptions and functional studies of changes of the cytokine milieu within the tumor microenvironment upon anti-angiogenic therapy could lead to targeting the upstream cause of resistance to treatment. In summary, understanding the biology of human TEMs may provide a novel, biologically relevant marker of tumor resistance to anti-angiogenic therapy and represent a previously unrecognized target of cancer therapy.

## MATERIALS AND METHODS

### Cell lines and culture

Human acute monocytic leukemia cell line THP-1 (American Type Culture Collection) was maintained in RPMI 1640 medium supplemented with 2 mM L-glutamine and 10% fetal bovine serum (SAFC Biosciences). When indicated, THP-1 cells were incubated under hypoxic conditions (1% O_2_, 5% CO_2_, 94% N_2_) and treated with IL4 and IL13 (20 ng/mL each; R&D System) for 48-72 hours to induce M2 polarization. U-87 MG human glioma cells (American Type Culture Collection) were cultured in minimum essential medium supplemented with 10% fetal bovine serum. GSC20 glioma stem cultures (generous gift by Dr. Frederick Lang, M.D. Anderson Cancer Center) were established from a human glioblastoma multiforme (GBM) surgical specimen and maintained as described previously [41]

### Animal studies

U-87 MG cells (5 × 10^5^) were implanted in the caudate nucleus of 6- to 8-week-old athymic nude mice (Harlan Sprague Dawley) by the screw-guided method as previously described [25]. The animals were then randomly separated into one of three treatment groups (10-15 mice per group): aflibercept (25 mg/kg s.c. twice weekly for a total of 3 weeks or 6 weeks, from day 10 after cell implantation), bevacizumab (10 mg/kg i.p. twice weekly for 6 weeks, from day 5 after cell implantation), or TMZ (7.5 mg/kg/day i.p., on days 4-8 and 18-22 after cell implantation). Either phosphate-buffered saline or hFc was administered as a control agent. Only those animals exhibiting generalized or local symptoms of disease were euthanized. Brains were fixed in 10% formaldehyde and embedded in paraffin. All animal studies were performed in the veterinary facilities of The University of Texas MD Anderson Cancer Center in accordance with institutional guidelines.

### Immunofluorescence analysis

Paraffin-embedded 5-μm tissue sections were deparaffinized and subjected to graded rehydration prior to staining procedures. For double immunofluorescence studies, the tissue sections were subjected to heat-induced epitope retrieval (10 mM citrate buffer, pH 6.0) and blocked with 10% serum. Endogenous biotin and streptavidin were blocked using avidin/biotin and streptavidin/biotin kits (Vector) according to the manufacturer's instructions. Overnight staining at 4°C with antibodies against Tie2 (C-20, diluted 1:100; Santa Cruz), MMP9 (AF911, diluted 1:50; R&D Systems), mouse Arg1 (V-20, diluted 1:1,000, Santa Cruz). Bound antibodies were detected by incubation with biotin-conjugated secondary antibody and DyLight 549 streptavidin (diluted 1:1,000; Vector). When required, the fluorescence signal was amplified with biotinylated anti-streptavidin antibody (diluted 1:500; Vector) followed by additional Dylight 549 strepavidin. Sections were incubated overnight at 4°C with Alexa Fluor 488-conjugated secondary antibody (diluted 1:1,000; Invitrogen).

Staining for F4/80 was used to detect microglia/macrophages in tumor sections. Heat-induced epitope retrieval was followed by 10 minutes of incubation with 20 μg/mL proteinase K (Sigma-Aldrich) at room temperature. Endogenous peroxidase activity was blocked with 3% H_2_O_2_. Blocking reagent from the Perkin Elmer tyramide signal amplification-plus fluorescein kit was used per the manufacturer's instructions. Incubation with an anti-F4/80 antibody (CI:A3-1; diluted 1:400; Serotec) was done overnight at 4°C and followed by 30 minutes of incubation with biotinylated secondary antibody and peroxidase-conjugated avidin (ABC Elite Kit; Vector). Sections were then incubated with fluorescein isothiocyanate-conjugated tyramide reagent for 5 minutes.

We also stained for Iba1 to detect microglia/macrophages in tumor sections. Standard antigen retrieval procedures and blocking with 10% serum were followed by incubation with an anti-Iba1 antibody (019-19741; diluted 1:1,000; Wako) overnight at 4°C. Afterwards, slides were incubated with Alexa Fluor 488-conjugated antibody (diluted 1:1,000, Invitrogen). Fluorescent-labeled slides were stained with 4',6-diamidino-2-phenylindole (DAPI; Sigma), mounted with fluorescence mounting medium (Dako), and examined under a fluorescent Zeiss Axiovert 200M microscope, an Olympus Fluoview FV1000 confocal microscope, or both.

For immunofluorescence analysis, 10 pictures from each experimental group were acquired under a 20x objective lens (HPF, high power field). The number of Tie2^+^, F4/80^+^, and Tie2^+^/Iba1^+^ cells was quantified in a double-blind manner by two different researchers.

### Immunohistochemical analysis

To perform immunohistochemical analysis, we quenched endogenous peroxidase activity with 3% H_2_O_2_. Sections were blocked with 10% rabbit serum, incubated with anti-MMP9 antibody (M-17; diluted 1:100, Santa Cruz) overnight at 4°C, and then incubated with biotinylated secondary antibody followed by peroxidase-conjugated avidin (ABC Elite kit, Vector) for 30 minutes each. The reaction was developed using stable 3,3′-diaminobenzidine (Research Genetics). Then, the sections were counterstained with Harris hematoxylin and mounted with Cytoseal 60 (Thermo Scientific). Images were captured using a bright-field microscope (Zeiss Axioskop 40).

### Flow cytometry and isolation of TEMs

To measure the expression level of Tie2, 5 × 10^5^ THP-1 cells were incubated with a reagent that blocks the cell-surface Fc receptor (120-000-442, Miltenyi Biotec) and exposed to phycoerythrin-conjugated mouse anti-human Tie2 antibody (FAB3131P, R&D Systems) and fluorescein isothiocyanate-conjugated mouse anti-human CD11b antibody (ab25827-100, Abcam). Mouse immunoglobulin G isotypes were used as controls. Samples were run on a flow cytometer (FACSCalibur, Becton Dickinson) and analyzed using CellQuest software. To isolate Tie2^+^ and Tie2^-^ subpopulations, THP-1 cells were incubated with IL4 and IL13 (20 ng/mL each, R&D Systems) under hypoxic conditions for 48-72 hours. Then cells were stained with phycoerythrin-conjugated mouse anti-human Tie2 antibody as described above. Tie2^+^ and Tie2^-^ cells were sorted with a FACSAria cell sorter (Becton Dickinson) and cultured for further experiments.

PBMCs were prepared from healthy blood donors (Gulf Coast Regional Blood Center, Houston, TX) by centrifugation on a Ficoll-Plaque Plus (GE Healthcare) density gradient as previously described [42]. CD14^+^ monocytes were purified from PBMCs by positive selection using CD14 MicroBeads (120-000-305, Miltenyi Biotech) per the manufacturer's instructions. The monocyte-enriched population was then double stained with anti-human allophycocyanin-conjugated CD14 antibody (FAB3832A, R&D Systems) and anti-human phycoerythrin-conjugated Tie2 antibody (FAB3131P, R&D Systems) (both, diwtion 1:10) in the presence of cell-surface Fc receptor-blocking reagent (Miltenyi Biotec). The suspension of stained cells was then subjected to flow cytometric analysis and cell sorting (FACSAria, Becton Dickinson) to obtain non-TEMs (CD14^+^/Tie2^-^ cells) and TEMs (CD14^+^/Tie2^+^ cells).

### Matrigel invasion assay

For the invasion assay, Tie2^+^ and Tie2^-^ THP-1 cells were isolated using a FACSAria cell sorter as described above and the condition medium was collected. Twenty four-well Transwell inserts (ThinCert-TC inserts, 8.0 μm; VWR) were coated with 0.3 mg/mL basement membrane matrix (growth factor reduced, LDEV free; BD Matrigel, BD Biosciences) for 2 hours at room temperature. A total of 1 x 10^5^ U-87 MG or GSC20 cells was washed with serum-free medium, suspended in 250 μL of serum-free medium, and seeded onto Matrigel-coated transwells. The lower chambers of 24-well plates were filled with 500 μL of conditioned medium from Tie2^+^ and Tie2^-^ THP-1 cells. After 24h of incubation, procedure was performed as previously reported [41]. Briefly, the remaining cells on the upper surface were mechanically removed using a cotton swab. The cells under the chamber were fixed with methanol and stained with 0.5% crystal violet (Sigma-Aldrich) for 20 minutes. Invading cells were counted under an Axioskop 40 microscope (Zeiss) equipped with Zeiss AxioVision Release 4.2 software (200x objective lens).

### Gelatinase assay

Gelatinolytic activity in conditioned medium from THP-1 cells or in PBMCs obtained from human peripheral blood was measured using the ENZCheck gelatinase/collagenase assay kit (Invitrogen) according to the manufacturer's instructions. Conditioned medium was collected as described above. Briefly, 100 μL of conditioned medium from similar numbers of cells were incubated with 12.5 μg/mL DQ gelatin (Invitrogen) for 2 hours at room temperature. Fluorescence activity resulting from digestion of DQ gelatin substrate was measured at 495 nm for absorption and 515 nm for emission wavelength.

### ELISA

We measured the level of MMP9 and MMP2 secretion in conditioned medium from CD14^+^Tie2^+^ and CD14^+^Tie2^-^ monocytic cell populations sorted from buffy coats from healthy human blood donors (Gulf Coast Regional Blood Center, Houston, TX). We used human MMP9 and MMP2 Quantikine ELISA kits (R&D System) for quantification according to the manufacturer's protocol.

The Tie2 phosphorylation level in THP-1 cells exposed to normoxia or hypoxia in the presence or absence of IL4 and IL13 was determined using the human phospho-Tie-2 (Y992) cell-based ELISA (R&D) as per the company's procedure. Briefly, we measured the phosphorylated Tie-2 (Y992) level in whole THP-1 cells grown on 96-well plates and exposed to normoxia or hypoxia in the presence or absence of IL4 and IL13. The cells were fixed, and a double immunoenzymatic labeling procedure was used to detect phospho-Tie-2 (Y992) and total Tie2 levels. Two secondary antibodies labeled with either horseradish peroxidase or alkaline phosphatase and two spectrally distinct fluorogenic substrates for horseradish peroxidase and alkaline phosphatase were used for detection. The fluorescence level of the phospho-Tie2 protein was normalized to that of total Tie2 in each well to correct for well-to-well variations.

### Zymography assay

THP-1 cells were cultured under either normoxic or hypoxic conditions, with or without IL4 or IL13, for 48 hours. During the last 24 hours the cells were cultured with medium plus 0.2% fetal bovine serum before the conditioned medium was collected. Conditioned medium (10-30 μL) from similar numbers of cells under the indicated treatments was loaded onto Novex 10% zymogram (gelatin) gel (Invitrogen). Electrophoresis was performed according to the manufacturer's instructions and followed by incubation with 2.5% (W/V) Triton X-100 for 30 minutes at room temperature. Then, the gel was equilibrated with 1x Novex zymogram renaturing buffer (Invitrogen) for 30 minutes and then incubated in 1x Novex zymogram developing buffer (Invitrogen) for 5 hours at 37 °C. The gel was stained with 0.5% Coomassie brilliant blue to visualize proteolytic active bands. The density of protease bands was analyzed using Quantity One software (Bio-Rad Laboratories).

### Statistical analysis

For quantitative data analysis, the results were plotted as the mean ± SD. Statistical analysis was performed using GraphPad Prism version 6.0 (GraphPad Software). Statistical significance was determined using Student *t*-test or correlation analysis computing Pearson's coefficient. *P* < 0.05 was considered statistically significant.

## SUPPLEMENTARY FIGURES


