# Supplementary material for: Anti-vascular endothelial growth factor therapy-induced glioma invasion is associated with accumulation of Tie2-expressing monocytes
Source: Oncotarget. 2014 Apr 11;5(8):2208–20. doi: 10.18632/oncotarget.1893 (PMC4039157; doi:10.18632/oncotarget.1893)
Supplement: Supplementary file 1 [file oncotarget-05-2208-s001.pdf]

Anti-vascular endothelial growth factor therapy-induced glioma invasion is associated with accumulation of Tie2-expressing monocytes

Supplementary Material

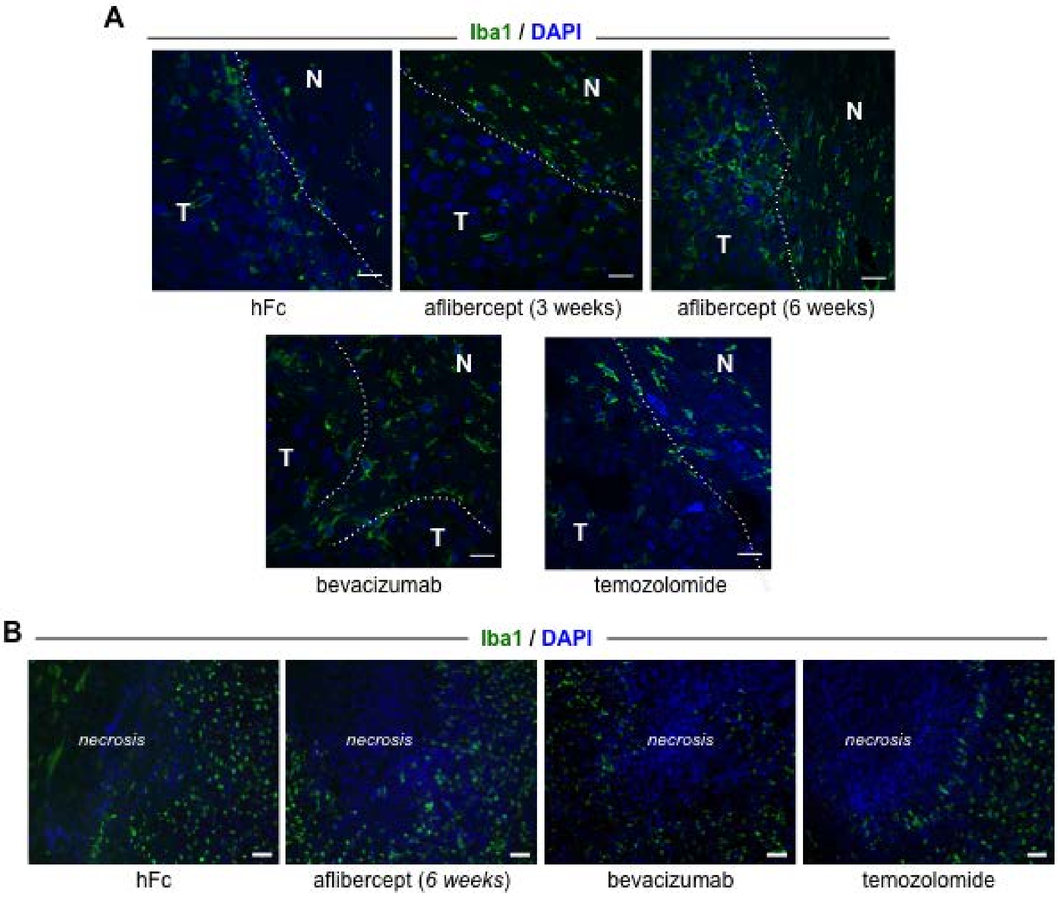

Supplemental Figure S1: Accumulation of tumor-infiltrating microglia/macrophages at the tumor/normal brain edge in brain sections of glioma-bearing mice treated with anti-VEGF agents. (A) Tumor sections from hFc-, aflibercept-, bevacizumab-, or TMZ-treated mice were stained for the Iba1 marker (green). DAPI was used for nuclear staining (blue). Infiltration of microglia/macrophages at the tumor/normal brain interface was strongest in the invasive tumors of mice treated with aflibercept (6 weeks) or bevacizumab. Dashed line indicates the border between tumor (T) and normal brain (N) tissues. Scale bar = 20 µm. (B) Representative images of $Iba1^+$ cells in surrounding necrotic tumor areas in brain sections from animals treated with hFc, aflibercept, bevacizumab, or TMZ. No notable differences in the infiltration of microglia/macrophages were observed. DAPI was used for nuclear staining (blue). Scale bar = 50 µm.

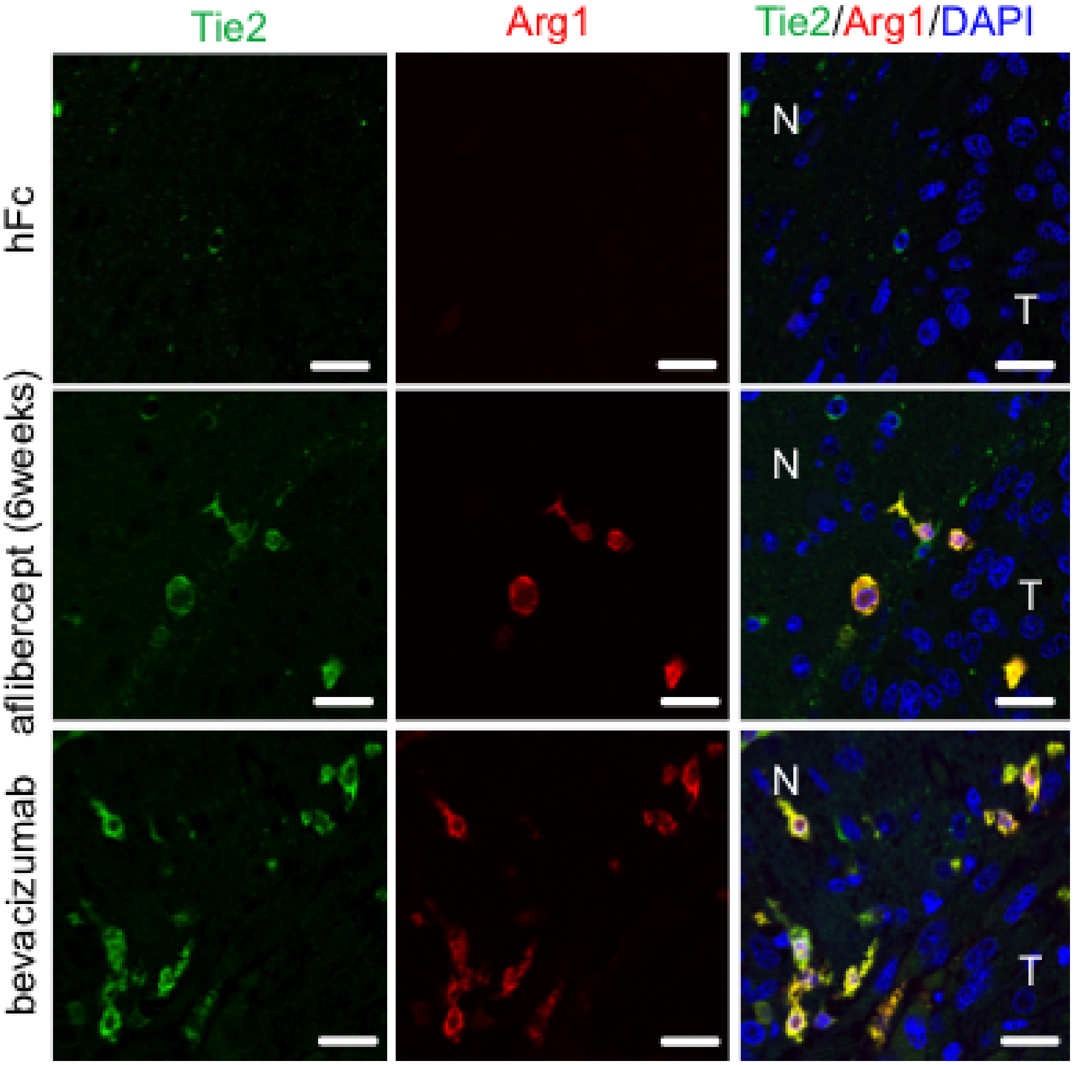

Supplemental Figure S2: Tie2$^+$ cells at the invasive edge of gliomas expressed Arg1 after anti-VEGF treatment. Representative confocal microscopy images of Tie2 (green) and Arg1 (red) immunofluorescence of brain sections from hFc-, aflibercept (6 weeks)- or bevacizumab-treated mice. DAPI was used for nuclear staining (blue). Tie2 and Arg1 immunostaining were co-localized. Note the increased number of Tie2$^+$Arg1$^+$ cells at the tumor edge in animals treated with anti-VEGF agents. N, normal tissue; T, tumor. Scale bars = 20 μm.

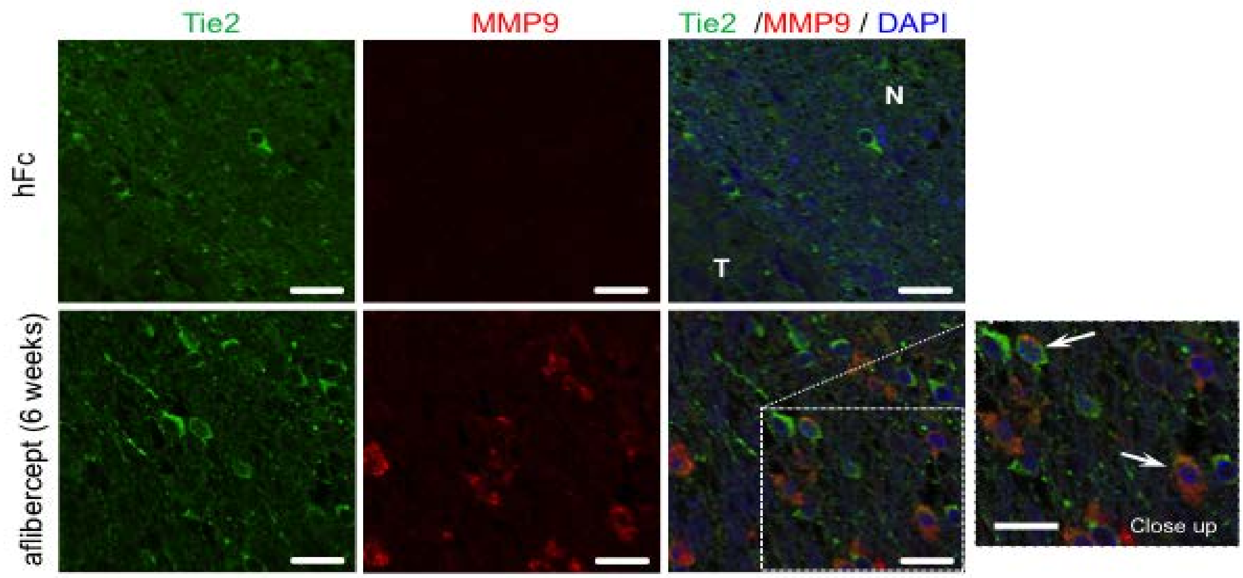

Supplemental Figure S3: After anti-VEGF treatment, Tie2$^+$ cells at the invasive edge of gliomas expressed MMP9. Representative confocal microscopy images of Tie2 (green) and MMP9 (red) immunofluorescence of brain sections from mice treated with hFc or aflibercept (6 weeks). DAPI was used for nuclear staining (blue). White arrows indicate cellular co-localization of Tie2 and MMP9 expression. Note the increase of Tie2$^+$MMP9$^+$ cells at the tumor edge in animals treated with aflibercept. N, normal tissue; T, tumor. Scale bars = 20 µm.
